# Development and initial psychometric testing of a new instrument for assessing empathetic clinical gaze among medical students

**DOI:** 10.3389/fmed.2025.1697526

**Published:** 2026-01-15

**Authors:** Lena Halawi, Peter Hagell, Atika Khalaf

**Affiliations:** 1 Faculty of Health Science, Kristianstad University, Kristianstad, Sweden; 2 Hind Bint Maktoum College of Nursing and Midwifery, Mohammed Bin Rashid University of Medicine and Health Sciences, Dubai Health, Dubai, United Arab Emirates

**Keywords:** clinical empathy, medical education, medical students, physician-patient relationship, psychometrics, scientific medical gaze

## Abstract

**Introduction:**

The traditional scientific medical “gaze” often promotes a detached form of clinical empathy that hinders effective communication between physicians and patients, neglecting the emotional dimensions of patient experiences. Although empathy involves both cognitive and emotional components, there is a notable lack of assessment tools that treat clinical empathy as a unified attribute. To address this gap, the concept of empathetic medical gaze (EMG) is proposed and an associated scale was developed, designed to assess medical students’ and practitioners’ genuine interest in patients through emotional attunement.

**Methods:**

An initial study was conduct to test the basic psychometric properties of the EMG scale. A 20-item instrument was created and assessed among 251 medical students in Sweden.

**Results:**

Item and exploratory factor analyses yielded a 16-item scale, which demonstrated good reliability (α = 0.87), corrected item-total correlations ranging from 0.37 to 0.60 and a single factor solution, thereby supporting unidemensionality and the use of a total empathetic clinical gaze score.

**Discussion:**

Future research is needed to explore refinement of the omitted items, test additional psychometric properties (e.g., test-retest stability, external construct validity) and apply more advanced psychometric methods, such as Rasch measurement theory, to further improve the understanding and assessment of this empathetic attribute.

## Introduction

William Osler’s assertion that the practice of medicine is an art, not a trade (1) challenges us to reconsider how we understand empathy in healthcare. Empathy, in a clinical context, refers to the capacity to recognize, understand, and resonate with a patient’s emotions and experiences, encompassing both cognitive and affective dimensions. In this context, this study proposes a new conceptual framework, the empathetic clinical gaze (ECG), which aims to harmonize emotional engagement with scientific rigor in patient care. This framework allows looking beyond the clinical objectivity prevalent in today’s medical practice, striving for a more holistic approach that honors the subjective experiences of patients while integrating the insights provided by scientific knowledge.

Historically, medical practice has grappled with a tension between humanistic approaches, which emphasize compassionate integration of scientific knowledge with genuine personal connection (2), and the “medical gaze” described by Foucault (3). Foucault’s concept highlights a shift toward a scientific framework that prioritizes disease over individual patient experiences, fostering clinical detachment. This “scientific medical gaze” objectifies patients and reduces them to clinical objects for diagnosis and treatment, thereby overlooking their emotional and psychosocial dimensions (3).

This persistent mode of thinking cultivates a “detached concern,” a cognitive form of empathy emphasizing objectivity and emotional distance over empathetic engagement, which challenges healthcare professionals’ (HCPs) emotional attunement when traditional perspectives advocate for detachment. Empathy significantly enhances interpersonal interactions in clinical healthcare and contributes to healing beyond traditional medical treatments (4, 5). However, a unified theoretical framework for clinical empathy has been lacking, leading to confusion and inconsistency in its definition. Previous research often misrepresents empathy as a dichotomy between cognitive and affective/emotive forms, oversimplifying its complexity and favoring cognitive empathy, which may promote detached concern rather than genuine engagement (6–8). Clinical empathy specifically relates to the application of empathy within healthcare settings, characterized by a deeper engagement with patients that extends beyond clinical detachment. The concept of the scientific medical gaze traditionally promotes clinical empathy that is emotionally detached, undermining effective communication between physicians and patients. This approach often neglects the emotional aspects of patient experiences, limiting understanding and genuine care (6, 9). Halpern (6, 9, 10), Shapiro (7) argue for the necessity of affective empathy in conjunction with cognitive empathy to fully grasp patients’ perspectives and enhance medical care outcomes. The ECG is envisioned as an inclusive approach that fosters engaged curiosity, a mindset characterized by a genuine interest in the patient’s experiences and emotions, shifting away from the strictly scientific medical gaze and addressing the dichotomy in clinical empathy practices.

Empathy has been considered integrated with, and a prerequisite for person-centered care (PCC) (11). PCC prioritizes respect for patient preferences, values, and actively involves them in decisions about their own care (12). ECG, however, focuses specifically on the HCP’s internal perceptual stance. It concerns the quality of attention and genuine curiosity directed toward understanding the patient’s emotional reality. This distinction can be seen as being crucial because without such an empathetic perceptual foundation, even well-intentioned PCC practices can risk becoming merely procedural rather than truly responsive to individual patient needs.

Female medical students generally appear to exhibit higher empathy levels compared to their male counterparts (13, 14). Furthermore, research indicates a struggle to balance the scientific and artistic aspects of medicine during medical education. While some studies suggest increased or stable empathy levels (15, 16), most studies suggest a notable decline in empathy among students and residents over time (17–20). These contrasting findings can be due to various factors, including the nature of medical education programs. It has been argued that while often expressing commitment to values such as empathy and compassion, medical schools also promote a “tacit commitment to an ethic of detachment, self-interest, and objectivity” [(21), as cited in (7), p. 275]. This framework encourages the objectification of patients and reinforces a scientific viewpoint, leading to an alienation of physicians from their patients rather than fostering empathy. Consequently, cultural norms in medical education compel future physicians to suppress their emotions, further distancing them from the empathetic care expected in the practice of medicine (7). Thus, the scientific medical gaze appears to persist and give rise to a deficient clinical practice of empathy due to a sought-out ideal of detached concern, which shapes physicians’ perspectives, interpretation of medical information, and influences their attitudes and beliefs.

The lack of well-founded, quality-assured tools for assessing clinical empathy hinders systematic studies on patient safety and quality of care in healthcare training and practice. A systematic review by Hong and Han (22) identified 11 empathy assessment instruments. Among these, the highest quality instruments were the Consultation and Relational Empathy Measurement (CARE), Jefferson Scale of Empathy (JSE), and Therapist Empathy Scale (TES), with CARE and TES evaluating cognitive, emotional, and behavioral dimensions of empathy, while JSE focuses on the cognitive aspect (22). CARE is patient-reported, and TES relies on external observer ratings, whereas the JSE is HCP-reported (22). The JSE is widely used in medical education and practice. It conceptualizes clinical empathy through three factors; understanding, perspective-taking, and compassionate care, emphasizing a predominant cognitive definition of empathy (22, 23). Defining empathy as a predominantly cognitive trait assumes that emotions are unnecessary to understand what patients feel. However, the integration of emotions is crucial for facilitating proper understanding and communication and cannot be treated separately from cognition in the conceptualization of empathy (6, 9, 10). In contrast, the recently developed ELPIS scale (24) is an HCP self-report tool that assesses emotional empathy toward cancer patients, focusing specifically on affective responses in oncology contexts.

Halpern (6, 10), Shapiro (7) argue that true clinical empathy requires the integration of both cognitive understanding and affective emotional engagement, not as separate dimensions, but as a unified whole. To achieve this integrated empathy, a new “gaze” is proposed that is clinical and empathetic, i.e., an ECG that harmonizes the understanding of disease with the recognition of the patient as a person. This ECG is envisioned as an inclusive approach that fosters engaged curiosity, a mindset characterized by a genuine interest in the patient’s experiences and emotions. It shifts away from the strictly scientific medical gaze and draws from Halpern’s concept of clinical empathy as “engaged curiosity,” necessitating emotional attunement (6, 9). This study proposes that a clinical gaze grounded in engaged curiosity towards emotional attunement can effectively address the dichotomy associated with clinical empathy.

The proposed ECG incorporates theoretical commonalities from established empathetic frameworks, such as Halpern’s (6, 9, 10) concept of “engaged curiosity” and Charon’s (25) narrative medicine, which focuses on understanding patient stories. Furthermore, it integrates these empathetic approaches with Foucault’s (3) concept of the medical gaze. This synthesis creates a framework that maintains scientific rigor and objectivity while incorporating emotional engagement and an understanding of the patient’s reality. No previous framework has attempted to comprehensively bridge the traditional medical gaze with empathetic clinical approaches.

While existing instruments purporting to assess empathy address relevant dimensions of clinical empathy, none of the instruments are based on Halpern’s notion of “engaged curiosity,” i.e., a genuine interest in patients through emotional attunement. Thus, “engaged curiosity” can be seen as a precondition for an ECG, which can be interpreted as a perceptual attitude, committed to validating the patient’s experience by understanding their suffering and emotional needs in conjunction with illness. Thereby, an ECG derives from a genuine interest in the patient and is specifically concerned with the level of curiosity in one’s clinical “gaze” about what the patient is concerned about, non-verbal attunement, and the effort to imagine the patient’s experience. The notion of the ECG is an attempt to overcome the potential pitfalls of the taxonomy surrounding clinical empathy and the prevailing dichotomy that divides empathy into cognitive and emotional components.

To operationalize this ECG framework, we propose the ECG Scale (ECGS) to assess medical students’ and practitioners’ genuine interest in patients through emotional attunement. The introduction of the ECGS is prompted by several shortcomings in the existing definitions and tools for assessing clinical empathy. Traditional frameworks often oversimplify empathy by categorizing it into cognitive and affective/emotive components, creating a dichotomy that fails to capture the complexity of human emotions in healthcare settings. This division skews the priorities in clinical practice, as cognitive empathy tends to be emphasized over affective aspects, potentially contributing to a detached, mechanical approach to patient care. Existing assessment tools also fall short in their ability to address the depth of empathy that encompasses both emotional engagement and cognitive understanding. Most available instruments tend to neglect the vital emotional attunement necessary for comprehensive patient care. For example, while the JSE provides valuable insights into cognitive empathy, it overlooks the necessity of emotional connection and the nuance of patients’ experiences. Moreover, many existing tools, such as the CARE and TES, do not specifically incorporate the concept of “engaged curiosity” as a driving force behind empathetic interactions. This lack of focus on genuine curiosity and emotional engagement limits their effectiveness in evaluating clinicians’ ability to connect with patients on a deeper level. Furthermore, the ELPIS scale has a context-specific emotional focus, and akin to the aforementioned instruments; it does not assess the unified perceptual attitude of “engaged curiosity” that manifests through emotional attunement across clinical contexts. Additionally, it does not evaluate the general disposition toward emotional engagement as a mode of clinical perception applicable across medical specialties and patient populations.

Empathetic clinical gaze and the ECGS intend to address these gaps by proposing a holistic approach that integrates cognitive and emotional dimensions, fostering an attitude of engaged curiosity. This reflective stance encourages clinicians to actively consider patients’ emotional experiences, thus enhancing communication and understanding. By viewing the patient as a person with unique emotional needs rather than just a set of symptoms, ECG promotes a more comprehensive understanding of patient experiences. As such, it fills important gaps by advocating for a more integrated definition of clinical empathy, emphasizing emotional attunement, and reframing the clinician’s perspective to incorporate genuine curiosity. In parallel, the ECGS intends to evaluate the general disposition toward emotional engagement as a mode of clinical perception applicable across medical specialties and patient populations. By operationalizing ECG as a unified rather than multidimensional construct, the ECGS attempts to overcome the potential pitfalls of the rigid taxonomy surrounding clinical empathy. None of the existing instruments are based on Halpern’s notion of “engaged curiosity,” which configures itself as a genuine interest toward patients through emotional attunement.

Here we describe the development of an instrument intended to assess ECG and the results from the initial testing of its basic psychometric properties among medical students in Sweden.

## Materials and methods

### Design

The study was conducted as a cross-sectional psychometric study among Swedish medical students, using traditional psychometric methods based on classical test theory (CTT) (26, 27).

### Sample

The sample consisted of medical students from Swedish universities. Inclusion criteria were active students registered at a Swedish university, ranging from their first to final (sixth) year of study, who identified themselves as male or female. This criterion was informed by previous empirical studies highlighting the importance of the sex of medical students (28). In addition, participants needed to have clinical experience to contribute to the evaluation of the empathetic clinical gaze. Any medical students who did not meet these inclusion criteria were excluded from the analysis.

We aimed for a sample size of at least 200 participants to have a reasonable participant-to-item ratio. Twenty initial items (see below) and 200 participants yield a ratio of 10:1, which is commonly recommended for exploratory factor analysis (27).

### Data collection

#### Instrument development

The study was conducted in three stages: (1) concept development, (2) instrument development, and (3) psychometric testing.

#### Stage 1: concept development

This stage involved developing the desired concept and its definition as a basis for item development and was based on a review of the relevant literature concerning empathy, clinical empathy, and scientific medical gaze. We turned to Halpern’s (6, 9, 10) work where she explores the importance of emotional engagement in medical practice. Halpern argues for a shift from a purely objective clinical gaze to one that incorporates emotional and empathetic elements, which aligns with the essence of ECG. Additionally, Foucault’s explorations of the medical gaze delineate the traditional approach to medicine, which often objectifies patients (3). Foucault’s critique highlights the need for a paradigm shift toward a more empathetic understanding of patients. By synthesizing these perspectives, ECG positions itself as a comprehensive approach that blends the cognitive and affective dimensions of empathetic clinical practice, reinforcing the necessity for healthcare providers to engage meaningfully with their patients beyond clinical symptoms.

Empathetic clinical gaze was defined as a cognitively and affectively attached professional medical perception involving understanding the patient and recognizing the patient more as a person in a body, as opposed to just a biological body. It is exercised and expressed through the physician’s emotional attunement toward the patient’s experiences, feelings, and perspectives.

Empathetic clinical gaze differs from clinical empathy (as represented by, e.g., the JSE) and PCC in several ways (Table 1). Firstly, clinical empathy focuses on the clinician’s ability to understand and share the patient’s feelings, emphasizing emotional connection but often lacking a broader context. PCC advocates for treating patients as whole individuals, considering their preferences and values; however, it may not always emphasize the emotional dimension of physician-patient interactions. In contrast, ECG expands on these concepts by combining emotional attunement with a deeper professional understanding of the patient as a complete person, highlighting their experiences. Secondly, in terms of cognitive-affective integration, clinical empathy tends to lean more toward emotional responses rather than integrating these feelings within a professional framework of medical knowledge. PCC emphasizes patient experience but may not fully engage the clinician’s emotional involvement. ECG specifically merges cognitive assessments of medical conditions with an affective response to the patient’s narrative, leading to a sophisticated blend of empathy and clinical perception. Lastly, the nature of engagement differs across these constructs. Clinical empathy may remain within clinical interactions without fully addressing individual complexities. While PCC focuses on individualized care that respects and responds to the patient, it can sometimes lack depth in emotional connectivity. ECG promotes an enriched relational dynamic wherein HCPs actively engage with the patient’s lived experiences, fostering a deeper understanding of health concerns within personal contexts. ECG is not suggested to replace clinical empathy and PCC but to enrich these frameworks by integrating cognitive and emotional dimensions in a more comprehensive manner, allowing for a more profound and empathetic connection between physicians and patients. It acknowledges the emotional engagement that is central to clinical empathy while expanding this understanding to include a nuanced perception of the patient as a whole person.

**TABLE 1:** Comparing empathetic clinical gaze (ECG) with related established frameworks.

Dimension	Empathetic clinical gaze (ECG)	Clinical empathy (e.g., the JSE)	Person-centered care (PCC)
Primary focus	Emotional attunement through engaged curiosity	Cognitive understanding of patient perspective	Shared decision-making and patient autonomy
Theoretical basis	Integration of Foucault’s medical gaze with empathetic frameworks	Perspective-taking and emotional recognition	Personhood, patient rights and collaborative care models
Core components	Genuine interest, emotional resonance, narrative understanding	Perspective-taking, compassionate care, standing in patient’s shoes	Respect for patient values, shared decision-making, individualized care
Assessment focus	Authentic emotional engagement and curiosity	Empathetic attitudes and behaviors	Patient involvement and care coordination

#### Stage 2: instrument development

Iterative item development resulted in 20 statements that were deemed relevant and consistent with the definition of ECG. This process involved systematically drafting, reviewing, and refining items through brainstorming sessions, literature reviews, and discussions among researchers and experts in the field.

As facets of the ECG, it was assumed that emotional attunement and engaged curiosity can manifest in observable ways in clinical encounters, reflecting a HCP’s engagement with the patient’s experiences. Such manifestations are not merely internal states but translate into specific actions and perceptual orientations in clinical care. Emotional attunement appears in clinical behavior through active attention to patients’ non-verbal emotional signals, explicit consideration of how patients’ feelings influence their physical symptoms and treatment responses, and deliberate efforts to grasp the patient’s illness experience. This dimension underscores the importance of recognizing patients as whole individuals, ensuring that their emotional states are integrated into clinical assessments and care decisions. Engaged curiosity manifests as a genuine interest in what concerns the patient beyond symptoms, a willingness to imagine the patient’s perspective in their unique context, and a recognition that understanding patients’ emotions can improve outcomes. The ECGS operationalizes these concepts through items that assess perceptual attitudes and beliefs about the role of emotional engagement in clinical practice. The items evaluate how medical students/clinicians perceive and approach emotional cues, whether they believe considering a patient’s feelings is important for diagnosis and treatment, and their propensity to explore the patient’s broader life context beyond just their medical condition

The preliminary 20-item questionnaire was pilot-tested using a variation of the Delphi method to examine clarity, face validity, and content validity of the suggested items (29). The intention was to implement a condensed version of the Delphi technique, utilizing a single round of assessment instead of three (29). This method involved soliciting feedback from a panel of experts to thoroughly review the survey instrument. Panelists were chosen based on key factors: a minimum of 5 years of relevant experience in healthcare-related fields, established work in empathy research or questionnaire methodology/psychometrics, and active involvement as healthcare providers. The panel included a clinical and academic physician, three clinical and academic nurses experienced in questionnaire development, and one psychologist specializing in empathy research. This diverse expertise ensured feedback was well-informed and pertinent.

For face validity, experts evaluated the completeness, appearance, and wording of the instrument and suggested modifications. For content validity, they assessed item relevance and clarity concerning the empathetic clinical gaze, recommending changes as needed. These validity assessments led to editorial suggestions for revised item wording. The experts’ feedback also highlighted concerns regarding the use of seven response categories, expressing skepticism about respondents’ ability to reliably distinguish between all options. Specifically, the “Neutral” category often fails to provide meaningful distinctions and tends to serve as a catch-all. Thus, experts suggested that a four- or five-category scale would be more effective. This aligns with research by Johnson and Morgan (30), who emphasize that too many response categories can complicate decision-making and increase potential measurement error. This collective input led to implementing a four-point response to capture respondent attitudes accurately. By eliminating the neutral midpoint, this format encouraged respondents to express a definitive stance on each item, promoting more decisive responses and enhancing data interpretation. This yielded a summed total score between 20 and 80, with higher scores indicating higher ECG levels.

The resulting modified instrument version was then pretested on a small sample of medical students (*n* = 20). In addition to responding to the items, participants were asked to comment on and suggest improvements regarding item clarity and relevance. This yielded additional minor revisions, resulting in the instrument version being brought forward to stage 3 (psychometric testing).

#### Stage 3: psychometric testing

A convenience sample was recruited between March 14*^th^* and August 1*^st^*, 2022, via Facebook groups related to medical school students at seven Swedish universities. Brief information about the study was posted together with a URL that led the participants to a detailed description of the study and a consent statement where participants indicated their consent to participate. Consenting participants were then directed to the online 20-item ECG instrument, complemented by questions regarding age, gender, year of medical studies, and experience of clinical patient encounters. One reminder was posted in the same Facebook groups 2 weeks after the initial post.

The sampling approach using Facebook groups has notable limitations, primarily concerning selection bias. Participants recruited via this method may not be representative of the broader population of medical students, as those who engage on social media may differ in motivation or demographics. Specifically, students who are less active on social media, or from institutions with less engagement online, may be underrepresented, leading to a skewed sample. Individuals who are more active on social media tend, on average, to be more socially engaged (e.g., higher extraversion, greater bridging social capital). Thus, they may show different baseline levels or forms of empathy than less active peers, with adults showing mixed or negative associations. Online (virtual) empathy correlates with, but is not identical to, offline empathy (31, 32). To enhance representativeness, the study targeted participants from seven different Swedish universities, which helped capture a wider array of experiences and backgrounds. Additionally, the inclusion of demographic questions, such as year of study and gender, allowed for an analysis of how well the sample reflects the entire medical student population across these dimensions. The reminder posted 2 weeks after the initial recruitment also aimed to increase overall participation, potentially capturing those who may have previously overlooked the invitation.

### Ethical considerations

The study was approved by the Health Sciences Ethics Council at the Faculty of Health Sciences, Kristianstad University (Dnr: U2022-2.1.12-2110). In addition, the study emphasized data privacy, informed consent, and confidentiality throughout the online data collection process. Participants were provided with a clear consent statement detailing the nature of the study, its purpose, and their rights as participants. This transparency ensured that individuals were fully aware of what their participation entailed. Data collected from participants was anonymized, helping to protect personal information. By prioritizing these ethical standards, the study adhered to the recommendations and guidelines of the Helsinki Declaration for data collection among human participants.

### Data analysis

Psychometric analyses were conducted according to CTT (26, 27, 33) regarding the following psychometric properties: (1) data quality, (2) scaling assumptions, (3) acceptability, (4) internal consistency reliability and (5) unidimensionality. Data were analyzed using IBM SPSS version 29 and FACTOR version 12.04.05^1^ (34).

Data quality was examined to determine to which extent the EMG instrument can be administered successfully to the target sample (26, 33). This was done by calculating the percentage of missing data for individual items and the percentage of complete responses to all items, with the recommended criterion of missing item responses being <10% (26).

Scaling assumptions were examined to determine the legitimacy of summing item scores into a total score (26, 33). Recommended criteria include roughly similar response category frequency distributions, roughly similar item mean scores and SDs, and corrected item-total correlations (item-to-total score of remaining items correlations) > 0.30–0.40 (26, 35).

Acceptability was examined to determine whether the distribution of participant scores coincided with the coverage of the scale (26, 33). Recommended item level acceptability criteria are roughly equivalent response category endorsements, low maximum endorsement frequencies, and floor- and ceiling effects < 75%. Acceptability of total scores is considered supported if total scores cover the full possible score range, have a mean value close to the possible score midpoint, skewness between −1 and +1, and if floor- and ceiling effects do not exceed 15%–20%.

Reliability was assessed by coefficient alpha, which assesses to what extent the items are interrelated by contrasting the shared variance between items to the overall variance (33, 36). It is recommended that alpha should be >0.70 and preferably >0.80 (33). The influence on alpha of deleting one item at a time was also assessed; an increase in alpha when an item is deleted suggests problems with, e.g., construct conceptualization or multidimensionality. Inter-item correlations (item-to-item correlations) were also examined to assess score homogeneity, i.e., how related individual items are; an average inter-item correlation > 0.3 has been suggested as acceptable (33, 37). Finally, the standard error of measurement (SEM) was calculated (SD × √1-alpha) as an estimator of score precision expressed in the same unit as the total score (33).

Unidimensionality was tested by exploratory factor analysis (EFA) using parallel analysis (PA) with minimum rank factor analysis (MRFA) based on polychoric correlations (38–40). The number of factors was not preset but determined by PA according to the 95th percentile of 500 random correlation matrices (39). This included Bartlett’s test of sphericity (*p* < 0.001), the Kaiser–Meyer–Olkin measure of sampling adequacy (>0.81), and Mardia’s test of multivariate normality (*p* < 0.001) to determine the appropriateness of EFA based on polychoric correlations (38). The minimum salient factor loading was set at 0.32 (38–41). Confirmatory Factor Analysis (CFA) was not performed due to the study’s exploratory nature and the somewhat limited sample size.

## Results

A total of 255 students completed the ECGS, but two individuals did not meet the third inclusion criteria, as they identified themselves as non-binary. Another two participants were excluded for not having met the fourth inclusion criteria, leaving data from 251 participants available for analysis. Most (78%) were females and aged between 18 and 24 years (59%). The students represented all years of medical studies, with a predominance of year five students (Table 2).

**TABLE 2:** Participant characteristics (*n* = 251).

Characteristic	*n* (%)
**Gender**	
Female	195 (78)
Male	56 (23)
**Age**	
18–24 years	148 (59)
≥24 years	103 (41)
**Medical school year**	
Year 1	50 (20)
Year 2	30 (12)
Year 3	30 (12)
Year 4	44 (18)
Year 5	67 (27)
Year 6	30 (12)

Data quality was excellent as there were no missing responses to any of the 20 items (Table 3).

**TABLE 3:** Item endorsement frequencies of the empathetic clinical gaze instrument among medical students in Sweden (*n* = 251).

Description of the content of items (abridged)	Item score distribution, *n* (%)				
	1^a^	2	3	4	Missing
1. (R) The perceived importance of biochemical factors	82.1	14.3	3.2	0.4	0
2. (R) The perceived importance of physical/psychological factors	58.6	31.1	9.6	0.8	0
3. Perceptual attitude regarding patient’s feelings’ impact on care quality	0.4	1.6	37.5	60.6	0
4. Perceptual attitude regarding patient’s feelings’ impact on wellbeing	0	1.2	17.5	81.3	0
5. (R) Perceptual attitude regarding lack of patient-centeredness	4.4	38.6	53.0	4.0	0
6. Perceptual attitude regarding the importance of considering a patient’s perspective	1.2	8.0	40.6	50.2	0
7. Perceptual attitude regarding the importance of understanding a patient’s situation	1.6	11.6	40.2	46.6	0
8. The perceived impact of empathy on clinical outcomes	3.6	19.5	56.6	20.3	0
9. The perceived impact of empathy on diagnosing	9.6	30.3	49.0	11.2	0
10. (R) Perceptual attitude regarding physician traits in understanding patients	0	2.0	30.3	67.7	0
11. (R) Perceptual attitude regarding the disregard of patients’ emotions	64.5	29.5	5.6	0.4	0
12. Perceptual attitude regarding patients’ emotions and improved outcomes	0.8	4.4	46.2	48.6	0
13. Perceptual attitude regarding patients’ feelings being key in diagnosis	0.8	10.8	43.0	45.4	0
14. Perceptual attitude regarding patients’ feelings being key to treatment	0.4	0.4	25.5	73.7	0
15. The perceived importance of understanding patients’ non-verbal signals	0	2.0	23.5	74.5	0
16. The perceived importance of thinking like a patient for the improvement of outcomes	6.0	32.3	47.8	13.9	0
17. The perceived importance of empathy as a quality	0.4	2.0	10.4	87.3	0
18. The perceived improvement on clinical competence procured through empathy	2.4	12.0	43.8	41.8	0
19. Perceptual attitude regarding family members’ feelings being key in diagnosing	8.8	25.5	49.0	16.7	0
20. Perceptual attitude regarding patients’ feelings being key to treatment outcomes	4.0	6.0	37.8	52.2	0

^a^1, Strongly disagree; 2, Somewhat disagree; 3, Somewhat agree; 4, Strongly agree. (R), reverse scored.

Scaling assumptions were not fully met by the 20-item instrument. All response categories were used for all items, except for items 4, 10, and 15 (Table 3). Most items exhibited corrected item-total correlations > 0.30 (Table 4), except for items 1, 2, 5 and 10 (−0.30 to 0.18).

**TABLE 4:** Scaling assumptions, acceptability, reliability and unidimensionality of the empathetic clinical gaze scale among medical students in Sweden (*n* = 251).

Psychometric property	20 items	16 items^a^
**Scaling assumptions**		
Item mean scores, min-max^b^	1.34–3.84	2.62–3.84
Item SD, min-max	0.43–0.84	0.43–0.84
Corrected item-total correlation, min-max^c^	−0.30 to 0.60	0.37–0.60
**Acceptability**		
Possible score range, min-max (midpoint)	20–80 (40)	16–64 (32)
Total score, mean (SD)^d^	64.4 (6.3)	53.4 (6.2)
Total score, median (q1–q3)^d^	65 (61–69)	50 (46–54)
Total score, min-max^e^	32–77	22–64
Total score floor/ceiling effects, %^f^	0/0	0/1.2
Total score skewness^g^	−1.09	−1.16
**Reliability**		
Coefficient alpha (all items)^h^	0.82	0.87
Alpha if item deleted^i^	0.81–0.84	0.86–0.87
Inter-item correlation, mean (min-max)^j^	0.184 (−0.38 to 0.57)	0.298 (0.09–0.57)
Standard error of measurement^k^	2.65	2.23
**Unidimensionality^l^**		
Bartlett’s test of sphericity, *p*-value^m^	<0.001	<0.001
Kaiser-Meyer-Olkin MSA, overall (item level min-max)^n^	0.81 (0.47–0.90)	0.86 (0.78–0.91)
Mardia’s test of multivariate normality, *p*-value^o^	<0.001	<0.001
PA, F1/F2% variance explained (empirical)	45.1/11.6^p^	55/8.8^q^
PA, F1/F2% variance explained (simulated data)^r^	14.4/12.5^p^	18.4/15.9^q^
Communality (h^2^)^s^^,^ ^t^	0.46–1.0	0.56–0.98
F1 loadings (min-max)^t^	−0.44 to 0.80	0.48–0.79
Explained common variance^t^	46.1	56.9

*^a^*Excluding items 1, 2, 5, and 10.

*^b^*Item scores range from 1 to 4.

*^c^*Should be >0.30–0.40 (26, 33).

*^d^*Should be close to scale midpoint (26, 33).

*^e^*Should span most of the possible score range (26, 33).

*^f^*Should be ≤15%–20% (26, 33).

*^g^*Should be ≤±1 (26, 33).

*^h^*Should be ≥0.80 (33).

*^i^*Should not increase compared to coefficient alpha for all items (27).

*^j^*Should average > 0.3 [(33, 37)).

*^k^*Should be <1/2 SD (42).

*^l^*Item level exploratory factor analysis (minimum rank factor analysis) based on a polychoric correlation matrix.

*^m^*Should be <0.05 (38).

*^n^*Should be >0.8 for the whole instrument and ≥0.5 for items (34, 38).

*^o^*Non-significance suggests multivariate normality; *p* < 0.05 suggests use of a polychoric correlation matrix (38, 39).

*^p^*Based on 14 extracted factors, as suggested by the Ledermann bound [(40)].

*^q^*Based on 11 extracted factors, as suggested by the Ledermann bound [(40)].

*^r^*Based on the 95th percentile of common explained variance from 500 random polychoric correlation matrices obtained by permutation of the empirical data (38, 39).

*^s^*Should be >0.4 (38).

*^t^*From final unrotated single-factor solution. SD, standard deviation; q1–q3, inter-quartile range; MSA, measure of sampling adequacy; PA, parallel analysis; F, factor.

Acceptability assessments showed that total scores spanned most of the possible score range, but respondents tended to score toward the higher end, although floor- and ceiling effects were negligible (Table 4).

Reliability was acceptable, as indicated by a coefficient alpha of 0.82 (Table 4). However, alpha increased to 0.83–0.84 when items 1, 2, 5, and 10 were deleted. Furthermore, items 1, 2, 5, and 10 exhibited negative inter-item correlations in 36 out of 70 instances.

Unidimensionality testing of the 20-item instrument using EFA suggested one factor that accounted for 46.1% of the variance (Table 4). However, item 5 exhibited an inferior MSA value (0.47) and items 1, 2, 5 and 10 loaded <0.32 (−0.44 to 0.25). In accordance with the observed corrected item-total correlations (see above), this suggests problems with these items.

Taken together, the results reported above showed promise but analyses of scaling assumptions (corrected item-total correlations), reliability (coefficient alpha) as well as unidimensionality (EFA) suggested issues with items 1, 2, 5, and 10. Specifically, these items appeared to compromise the degree of conceptual homogeneity of the instrument by failing to operationalize the latent ECG variable in harmony with the remaining 16 items. These four items were therefore omitted, and the resulting 16-item instrument (possible total score, 16–64; higher scores = higher EMG levels) was re-analyzed. Results from these analyses showed improved scaling assumptions (corrected item-total correlations, ≥0.37), reliability (coefficient alpha, 0.87), and unidimensionality (factor 1 loadings, ≥0.48; common variance explained, 56.9%), while acceptability was largely unaltered (Table 4).

## Discussion

This study proposes the concept of ECG, which encompasses cognitive and emotional traits aligned with scientific medical gaze, and describes the development and initial testing of an instrument for assessing ECG, the ECGS. The results indicated that 16 out of 20 proposed scale items performed adequately to yield an interpretable unidimensional total score that meets recommended traditional psychometric criteria, demonstrating that the ECGS successfully operationalized the ECG construct.

To the best of our knowledge, the ECGS provides the first self-report measure that operationalizes clinical empathy as a unidimensional perceptual attitude grounded in engaged curiosity and emotional attunement, rather than treating cognitive and affective empathy as separate dimensions or focusing exclusively on one component. This study is effectively situated within the philosophical frameworks of Foucault (3), Halpern (6), bridging critical theory with psychometric development. The ECGS encapsulates an integrated approach that harmonizes disease understanding with patient recognition, addressing a significant challenge not directly tackled by existing instruments.

### Scale development and item refinement

Exploratory factor analysis and item analysis supported the unidimensionality of 16 items from the original scale. The findings indicated that four items (1, 2, 5, and 10) did not represent the same latent ECG variable as the remaining 16 items, thus suggesting that they should be omitted. Importantly, the omission of these four items does not narrow the scope of what the ECG construct assesses. Therefore, the scale remains robust in assessing the key aspects of an ECG, translating abstract philosophical concepts of emotional attunement and engaged curiosity into a unidimensional variable.

The omitted items (1, 2, 5, and 10) did not align with the core definition of the ECG for several reasons. First, items 1 and 2 emphasize aspects of the scientific medical gaze, focusing on objective clinical observations rather than the emotional and interpersonal connections vital to ECG. Another reason is potential ambiguity in wording, seen in items 5 and 10, compromising their ability to capture the nuanced emotional interactions central to the concept of ECG. Last but not least is the issue of reversed scoring presented in all four omitted items. The items had to be reverse scored, i.e., re-coded to have the same directional relationship with the construct as the other items on the scale. It is known that reverse scoring can have negative consequences by, e.g., causing a reduction in internal consistency due to low item total correlation (42), which is evident for ECGS items 1, 2, 5, and 10. Another related consequence is that reverse scored items may influence the factorial structure, thereby impacting results regarding dimensionality (42). This study found evidence of both. By omitting these items, clarity and precision of the ECGS was enhanced, thereby strengthened its alignment with the core definition of ECG and ensuring that it assesses the critical emotional and relational components of empathy necessary for effective patient care. However, the omitted items may warrant further review, particularly regarding their wording, to allow for final conclusions regarding their role in the scale.

These experiences illustrate an important, albeit not novel methodological aspect of scale development. While reverse-scored items traditionally are included to control for acquiescence bias, their inclusion may come at significant costs, as the inherent difference in wording may confuse respondents and represent different response processes (43), which outweigh their intended benefits. Our findings illustrate the importance of critically evaluating the necessity of reverse-scored items during initial development and conducting thorough pilot testing to assess their impact on, e.g., internal consistency and dimensionality.

### Practical applications in medical education and clinical training

The distinction between the conceptual underpinnings of the ECGS and JSE has practical implications for curricula and clinical training by emphasizing the integration of affective components alongside cognitive assessments. A key element of the ECGS is the concept of engaged curiosity, which underscores the importance of emotional attunement in the empathetic process. This may allow educators to foster not only the intellectual understanding of empathy but also the deeper engagement needed for effective patient interactions. In curricula development and evaluation, ECG (and the ECGS) could guide the development of training programs that prioritize emotional engagement, encouraging students to cultivate a genuine curiosity about their patients’ experiences and emotions. This creates an environment where future HCPs learn to connect on a personal level, enhancing their ability to understand and respond to patients’ needs. In clinical training, this focus on an ECG has the potential to lead to, e.g., the design of role-playing scenarios and reflective practices that simulate real-world situations, fostering skills in emotional intelligence and empathetic communication. Ultimately, by enriching training with a conceptualization of empathy that includes engaged curiosity, the ECG/ECGS shows promise for ensuring that HCPs are equipped not just with knowledge, but with the ability to approach patient care holistically, considering also emotional connection. This integrated approach has the potential to contribute to more compassionate and effective healthcare.

This integrated conceptualization is supported by the factor structure of the ECGS, which suggests a unidimensional rather than multidimensional structure. This is also in agreement with theoretical work proposing that engaged curiosity through emotional attunement is the sole effect indicator, whereas cognition, understanding, and communication can be seen as casual indicators (6, 9, 10).

To be integrated into the foundation of medical education and practice, empathy needs to be incorporated into the scientific medical gaze of medical students and professionals to strengthen focus on the person behind the patient. ECG and the ECGS described in this study offer initial steps toward an opportunity to improve understanding of the application of empathy to the scientific medical gaze, and to assess the level of ECG that is rendered. Pending further validation, the ECGS has the potential to assess the impact of training and could potentially serve as a tool to positively influence medical education and, in the longer term, promote PCC. This is particularly relevant since empathy has been considered intrinsic to successful PCC delivery (11). As such, the ECGS also has the potential to assess ECG of, not just, medical students and practitioners, but of other healthcare students and professionals as well. However, this will require further developmental work, as well as testing to ensure its equal applicability across these groups. Such efforts appear warranted since empathy and PCC concern all HCPs.

### Cognitive-affective integration: theoretical implications

The finding that the ECGS appears unidimensional suggests that the items represent a common variable. This variable is hypothesized to be a coherent behavioral manifestation of empathetic clinical practice. This aligns with Halpern’s (6, 9, 10) conceptualization of engaged curiosity as an integrated empathetic response where cognitive perspective-taking and emotional attunement function as interconnected rather than separate processes. The unidimensional structure suggests that respondents who demonstrate genuine interest in patients’ experiences (cognitive component) also exhibit emotional resonance with their perspectives (affective component), supporting the theoretical premise that these elements are integrated. However, there is a distinction between true cognitive-affective integration, as proposed by conceptual frameworks like Halpern’s engaged curiosity and clinical phenomenology, and strongly correlated but separate constructs. True integration would suggest that empathetic clinical practice cannot be meaningfully separated into cognitive and emotional components, whereas correlated constructs would imply that while these processes typically co-occur, they may differ in individual practitioners. Although unidimensionality is central in measurement, it remains a relative rather than an absolute matter, leaving conceptual arguments in the forefront, whereas statistics merely provides supporting or contradicting evidence. Thus, this study cannot make this distinction regarding the ECGS but additional evidence and conceptual inquiry are required.

### Methodological limitations

The primary purpose of this study was to develop items representing a new conceptualization of empathy in medical settings and to obtain initial indications of their psychometric feasibility. This iterative process involved concept development and item generation based on literature reviews, expert input, and small sample pre-testing, followed by initial psychometric evaluation. While the procedures and results reported here represent appropriate first fundamental steps in scale development, this section discusses potential limitations and areas for future work toward establishing the ECGS.

The decision to develop the scale using only binary individuals (male or female) aligned with previous research (28) that emphasized the relevance of biological sex. This may be questioned in terms of current inclusivity standards as well as threatening the external validity of the findings. However, given that only two persons were excluded based on this it is considered highly unlikely that it affected the findings. Our sample also consisted of mainly female participants. Although reflecting the demographic trend in Swedish medical schools (44), this may have affected observed scores since females typically report higher empathy levels than male students (45, 46), thereby potentially also influencing score variances and psychometric properties. However, minimal ceiling effects suggest acceptable targeting and argue against significant psychometric implications. Nevertheless, future studies would benefit from a more balanced sample, including persons identifying themselves as non-binary, to ensure broader representativeness.

The development and initial testing of the ECGS was conducted with a sample from Swedish universities, which may limit the generalizability of these findings across different cultural contexts. Empathy expression, medical education approaches, and patient-provider interactions can vary between cultures. In addition to translation, cross-cultural validation studies will therefore be needed before the ECGS can be applied in international settings. Future research should involve diverse samples from various countries to assess the instrument’s invariance and conceptual equivalence and measurement properties across different cultural backgrounds and healthcare systems.

This study focused on the internal validity of the ECGS, addressing the legitimacy of creating summed total scores, its internal structure (unidimensionality), appropriateness for the target sample and internal consistency reliability, which is considered fundamental for further testing (26, 27, 33). However, several psychometric properties remain untested. These include test-retest stability, responsiveness to change and external construct validity, which concerns associations with other relevant instruments, variables, and outcomes. The absence of evidence regarding such properties is a limitation. However, the current study was not designed for these purposes. Further research is needed to address these properties, e.g., external construct validity relative to other empathy scales where it, e.g., would be reasonable to expect moderate correlations (0.4–0.6) with cognitive JSE scores, and somewhat stronger correlations with patient-reported CARE scores (emotional empathy), and to reflect improvements following successful empathy training. Furthermore, modern psychometric approaches, such as Rasch measurement theory (RMT), should also be considered. As opposed to CTT-based methods, RMT offers a more nuanced understanding of rating scales by addressing aspects such as response category functioning, local independence, transformation of ordinal raw scores to linear measures, and differential item functioning. Such insights would contribute to refining the overall effectiveness of the instrument. Until more comprehensive psychometric evidence is available, the ECGS should be considered a preliminary instrument under development requiring further testing before implementation in educational or clinical contexts.

## Conclusion

The ECGS represents a pilot testing of the concept of clinical empathy in medical care as an ECG that integrates cognition and emotion through emotional attunement and engaged curiosity, highlighting that an ECG not only involves a deep emotional connection with patients but also an active interest and inquisitiveness about their experiences. This combination emphasizes that effective clinical empathy comes from understanding emotions while also being curious and seeking to learn more about patients’ perspectives.

While psychometric findings reveal that 16 of the initial 20 ECGS items collaboratively provide a reliable and interpretable unidimensional total score that meets fundamental traditional psychometric criteria. These findings represent initial development work and the resulting ECGS is not a ready-to-use instrument. Instead, findings are intended to inform subsequent iterations of scale development. Future research should aim to develop a more comprehensive conceptualized definition of ECG that is applicable across a range of healthcare students and professionals. This exploration should also incorporate further psychometric work, including RMT, to enhance understanding of the concept and refine the scale. Furthermore, future validation studies should prioritize gender-balanced sampling and cross-cultural validation to establish the robustness of the ECGS and ensure its applicability across diverse medical student populations.

## Data Availability

The raw data supporting the conclusions of this article will be made available by the authors, without undue reservation.

## Footnotes

1
https://psico.fcep.urv.cat/utilitats/factor/Download.html
